# Distinct Molecular Mechanisms Underlying Potassium Efflux for NLRP3 Inflammasome Activation

**DOI:** 10.3389/fimmu.2020.609441

**Published:** 2020-12-07

**Authors:** Ziwei Xu, Zi-mo Chen, Xiaoyan Wu, Linjie Zhang, Ying Cao, Pingzheng Zhou

**Affiliations:** ^1^Guangdong Provincial Key Laboratory of New Drug Screening, School of Pharmaceutical Sciences, Southern Medical University, Guangzhou, China; ^2^19th grade, Pharmacy Major, School of Pharmaceutical Sciences, Southern Medical University, Guangzhou, China; ^3^Department of Neurology, Nanfang Hospital, Southern Medical University, Guangzhou, China

**Keywords:** P2X7 receptor, pannexin-1, Gasdermin D, K2P channels, TWIK protein-related acid-sensitive potassium channel 2, THIK-1, inflammasome

## Abstract

The NLRP3 inflammasome is a core component of innate immunity, and dysregulation of NLRP3 inflammasome involves developing autoimmune, metabolic, and neurodegenerative diseases. Potassium efflux has been reported to be essential for NLRP3 inflammasome activation by structurally diverse pathogen-associated molecular patterns (PAMPs) or danger-associated molecular patterns (DAMPs). Thus, the molecular mechanisms underlying potassium efflux to activate NLRP3 inflammasome are under extensive investigation. Here, we review current knowledge about the distinction channels or pore-forming proteins underlying potassium efflux for NLRP3 inflammasome activation with canonical/non-canonical signaling or following caspase-8 induced pyroptosis. Ion channels and pore-forming proteins, including P2X7 receptor, Gasdermin D, pannexin-1, and K2P channels involved present viable therapeutic targets for NLRP3 inflammasome related diseases.

## NLRP3 Inflammasome

Inflammasomes are intracellular multiprotein complexes and core components of innate immunity (1–3). To date, the NOD-like receptor (NLR) family and the PYHIN family have been reported to form inflammasomes (4). These are composed of six NLR family proteins, including NLRP1, NLRP2, NLRP3, NLRP6, NLRC4, NLRP12, and two members of the PYHIN family, including AIM2 and IFI16 (5, 6).

**Figure 1:**
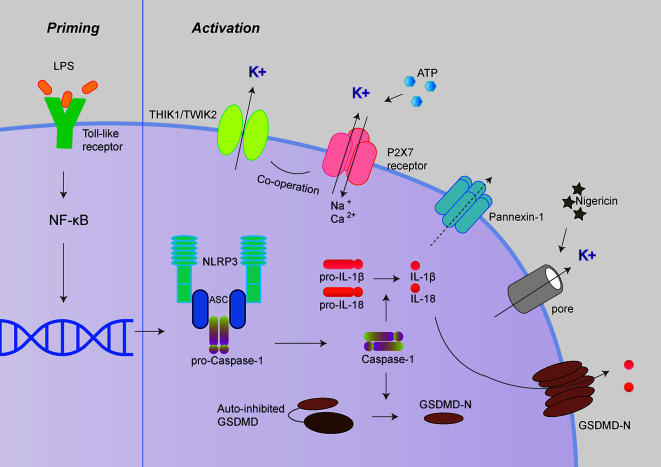
Canonical NLRP3 inflammasome activation. Canonical NLRP3 inflammasome activation includes two signals: priming and activation. The priming process leads to the expression of NLRP3, pro-IL-1β and pro-IL-18 could be provided by Toll-like receptors activation. The activation process promotes the assembly of inflammasome complexes through various PAMPs or DAMPs, including extracellular ATP, nigericin, and particulate matters. The activation of NLRP3 inflammasome cleaves pro-caspase-1 to active caspase-1, thereby cleaves pro-IL-1β and pro-IL-18 to produce mature IL-1β and IL-18. Besides, activated caspase-1 also cleaves GSDMD to release its N-terminal domain, which forms pores at the plasma membrane and mediates the release of mature IL-1β and IL-18. The P2X7 receptor, Pannexin-1, TWIK2, and THIK1 have been proposed to mediate potassium efflux during NLRP3 inflammasome activation under different circumstances.

Among various inflammasomes, NLRP3 inflammasome has been widely under investigation because of its most significant clinical relevance (7, 8). NLRP3 inflammasome consists of sensory protein NLRP3, adaptor protein ASC (the adaptor molecule apoptosis-associated speck-like protein containing a CARD), and effector protein caspase-1 (7, 8). Canonical NLRP3 inflammasome activation requires two steps: priming and activation. The priming process leads to the expression of NLRP3, pro-IL-1β, and pro-IL-18, which could be initiated by Toll-like receptors (TLR) ligands (9). The activation process promotes the assembly of inflammasome complexes, cleaving pro-caspase-1 to form active caspase-1, thereby cleaving pro-IL-1β and pro-IL-18 to release mature IL-1β and IL-18 (**Figure 1**).

**Figure 2:**
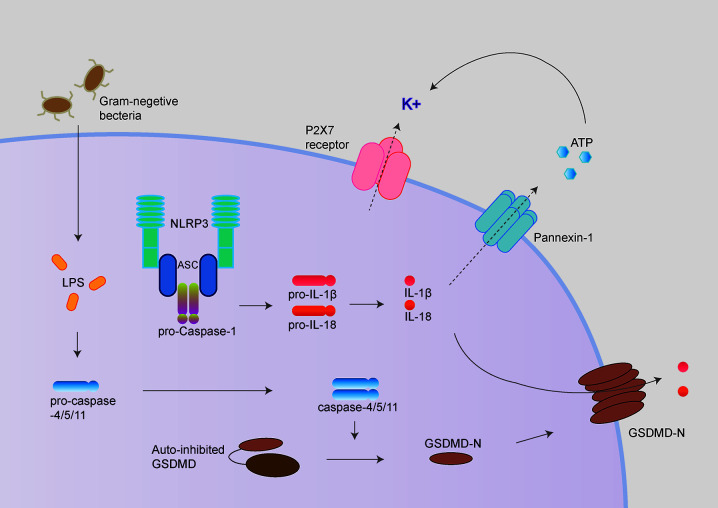
NLRP3 inflammasome activation following non-canonical inflammasome activation. The non-canonical inflammasome pathway is mediated by caspase-11 in mouse cell and caspase-4/caspase-5 in a human cell in response to cytoplasmic bacterial lipopolysaccharide (LPS). Caspase-4/5/11 cleaves GSDMD to initiate pyroptosis, thus leads to NLRP3 inflammasome activation. GSDMD and P2X7 receptor/Pannexin-1 have been proposed to mediate potassium efflux, which underlies the mechanism of NLRP3 inflammasome activation following non-canonical inflammasome.

Besides, activated caspase-1 also cleaves Gasdermin D (GSDMD) to release its N-terminal domain, which forms pores at the plasma membrane and induces a rapid, pro-inflammatory form of cell death termed “pyroptosis” (10–12). Intriguingly, the activation process of NLRP3 inflammasome could be provided by surprisingly various types of PAMPs (pathogen-associated molecular pattern) or DAMPs (danger-associated molecular pattern). These include extracellular ATP, pore-forming toxins (nigericin and maitotoxin, etc.), particulate matter (urate crystalline MSU, aluminum adjuvant, silica, and asbestos), and misfolded proteins related to neurodegenerative diseases (fibrillar Aβ protein; α-synuclein) (10–12). The dysregulated activation of NLRP3 inflammasome is closely related to various auto-inflammatory or chronic inflammations, such as gout, atherosclerosis, obesity, Alzheimer’s disease, Parkinson’s disease, and type 2 diabetes (13–15). Besides the canonical activation process, the non-canonical inflammasome pathway is mediated by caspase-11 in mouse cell or caspase-4/caspase-5 in a human cell in response to cytoplasmic bacterial lipopolysaccharide (LPS) (**Figure 2**) (16, 17).

Cytoplasmic LPS directly binds the caspase recruitment domain (CARD) of caspase-4/5/11, triggering caspase-4/5/11 cleaves GSDMD to initiate pyroptosis (18, 19). Caspase-11 mediated pyroptosis in response to cytosolic LPS is critical for antibacterial defense and septic shock in mice as demonstrated that GSDMD^–/–^ and caspase11^–/–^ mice could be protected against LPS-induced lethality (20, 21). Besides directly causing pyroptosis, the non-canonical inflammasome also promotes the canonical NLRP3 inflammasome to cause the maturation and release of IL-1β and IL-18 (19).

## Ion Channels and Pore-Forming Proteins Mediating Potassium Efflux During NLRP3 Inflammasome Activation

It has been well accepted that potassium (K^+^) efflux is both necessary and sufficient for NLRP3 inflammasome activation in most cases (22–25). First, a large reduction of intracellular potassium concentration was observed to activate the NLRP3 inflammasome by ATP, nigericin, and crystal molecules (23). Furthermore, incubation of primed macrophages in a K^+^-free medium was sufficient to trigger NLRP3 inflammasome activation (26). In contrast, NLRP3 inflammasome activation could be blocked by high concentrations of extracellular potassium (30-45 mM) (23, 26). Besides, AIM2 and NLRC4 inflammasomes activation was not affected by high concentrations of extracellular K^+^, indicating potassium’s specific role in modulating NLRP3 inflammasome (23–25).

Structurally diverse DAMPs/PAMPs employ distinct mechanisms to cause potassium efflux to activate the NLRP3 inflammasome. Firstly, the existing research mainly focuses on the molecular mechanism of potassium efflux during ATP-induced NLRP3 inflammasome activation. The P2X7 receptor, pannexin-1, and K2P channels have been reported to participate in the process above (24, 25, 27). Secondly, toxins such as nigericin directly promote potassium efflux by forming pores on the plasma membrane. NLRP3 inflammasome could also be activated following the non-canonical inflammasome or caspase-8 mediated pyroptosis, which also depends on potassium efflux (28–31). Controversially, GSDMD, and pannexin-1 have been proposed to mediate potassium efflux in the above process to activate NLRP3 inflammasome (19, 29, 30, 32, 33). This section will review the current knowledge about ion channels’ roles and pore-forming proteins mediating potassium efflux during NLRP3 inflammasome activation under different circumstances.

### P2X7 Receptor

The P2X7 receptor is an ATP-gated cation-selective channel widely expressed in various immune cells (34). At resting conditions, extracellular ATP concentration is at low levels (<10 nM/L), which will be massively increased to several tens or hundreds of μmoles/l within stressed or dying cells (35). The elevated extracellular ATP activates the P2X7 receptor, which then mediates potassium efflux and thus leads to NLRP3 inflammasome activation (34, 36–38).

Besides, the canonical NLRP3 inflammasome, P2X7 receptor, and pannexin-1 (see *Pannexin-1*) also have been reported to participate in non-canonical inflammasome (39) coordinately. It was reported that the activated caspase-11 cleaves pannexin-1 followed up by ATP release, which in turn activates the P2X7 receptor to mediate potassium efflux and NLRP3 inflammasome activation (39). Correspondingly, the P2X7 receptor ablation significantly reduced the mortality of mice and IL-1β secretion in peritoneal fluid in a sepsis mice model (39). However, this study is contradicted with studies by several other groups that we will discuss in the next section.

### Gasdermin D

Gasdermin D (GSDMD) has been identified as the executor of pyroptosis activated by caspase-1/4/5/11 in 2015 (19, 31, 40). Full-length GSDMD includes the N-terminal (GSDMD-N) and C-terminal repressor domain (GSDMD-C) interacting with each other in the absence of stimulation. This auto-inhibitory conformation is released upon efficient cleavage at a conserved glutamic acid residue (D276 in mouse and D275 in human GSDMD) caspase-1/4/5/11, dividing GSDMD into GSDMD-N and GSDMD-C. The generation of GSDMD-N allows it to insert into the plasma membrane and form large oligomeric pores, leading to IL-1β and IL-18 secretion and pyroptosis. Kayagaki et al. and Shi et al. reported that potassium pass through the pore-forming GSDMD, which further leads to NLRP3 inflammasome activation during non-canonical inflammasome activation (18, 19). Besides mediating pyroptosis and NLRP3 inflammasome activation, GSDMD was recently reported to restrain type I interferon response to cytosolic DNA by driving potassium efflux (41).

**Figure 3:**
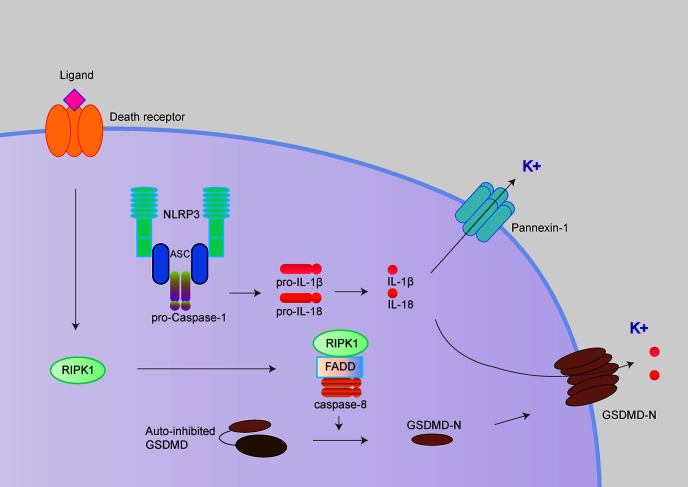
NLRP3 inflammasome activation following caspase-8 mediated pyroptosis. The NLRP3 inflammasome could also be activated following by caspase-8 mediated pyroptosis. The “apoptotic” caspase-8 cleaves GSDMD and further mediated potassium efflux leading to NLRP3 inflammasome activation in murine macrophages. In contrast, Chen et al. demonstrated the pannexin-1 but not GSDMD mediating potassium efflux contributed to NLRP3 inflammasome activation following caspase-8 mediated pyroptosis.

Caspase-8 has long been considered to play key roles in extrinsic apoptosis and suppress necroptosis by inhibiting RIPK1/RIPK3 and MLKL. More recently, three independent studies have demonstrated the “apoptotic” caspase-8 also could cleave GSDMD leading to pyroptosis-like cell death, further triggering NLRP3 inflammasome activation in murine macrophages (**Figure 3**) (29, 30, 32). It has been proposed that potassium efflux underlies NLRP3 inflammasome activation, followed by caspase-8 mediated pyroptosis. However, three groups disagree with the molecular mechanism underlying potassium efflux in the process above. Orning et al. and Sarhan et al. suggest that NLRP3 inflammasome activation is dependent on GSDMD-mediated potassium efflux based on delays in ASC oligomerization in GSDMD^-/-^ cells (29, 30). However, Chen et al. observed normal caspase-1 processing in GSDMD^-/-^ and/or GSDME^-/-^ (Gasdermin E; another member of Gasdermin protein) cells, which suggests NLRP3 inflammasome activation is not dependent on GSDMD or GSDME (32).

### Pannexin-1

The pannexin-1 is a non-selective, large-pore channel that releases potassium and nucleotides, including ATP (42, 43). Pannexin-1 is expressed in most cell types and functionally auto-inhibited by its cytoplasmic C-terminal domain. In response to apoptosis, the pannexin-1 channel can be functionally activated by caspase-3 mediated cleavage of the inhibitory C-terminal domain (44, 45).

The relationship between pannexin-1 and NLRP3 inflammasome is still controversial. By using pannexin-1 inhibitors or siRNA, Pelegrin et al. reported that pannexin-1 is responsible for IL-1β release upon NLRP3 inflammasome agonists ATP or nigericin (46–48). However, this channel was lately reported to be dispensable for canonical NLRP3 inflammasome activation using pannexin-1 knockout mice (49).

Together with the P2X7 receptor, pannexin-1 was also implicated in promoting pyroptosis and NLRP3 activation during non-canonical inflammasome activation (discussed in *P2X7 Receptor*) (39). In LPS-induced sepsis mouse models, the ablation of pannexin-1 significantly reduced mice mortality, which indicates the role of pannexin-1 in non-canonical inflammasome activation (39). This finding is at odds with the observation that caspase-11 drives NLRP3 inflammasome activation through GSDMD pores (18, 19, 40). A recent study further pointed out that pannexin-1 is dispensable for canonical or non-canonical inflammasome activation within pharmacological inhibition and two other macrophages strain with pannexin-1 ablation (33).

Interestingly, during the NLRP3 inflammasome activation following caspase-8 activated pyroptosis, Chen et al. observed that potassium efflux mediated by pannexin-1 but not GSDMD is critical for NLRP3 inflammasome activation following caspase-8 mediated pyroptosis (32, 33).

### K2P Channels

Two-pore domain potassium (K2P) channels comprise a major and structurally distinct subset of mammalian K+ channel superfamily, including fifteen K2P subtypes that form six subfamilies (TWIK, TASK, TRESK, TALK, THIK, and TRESK) (50, 51). K2P channels contribute to the background leak currents, responsible for maintaining the resting membrane potential in nearly all cells. They are regulated by various physical, chemical, and biological stimuli and implicated in multiple physiological processes. In recent years, significant roles of K2P channels for the activation of NLRP3 inflammasome and innate immunity have been gradually revealed (27, 52).

TWIK2 is a member of K2P channels, highly expressed in the gastrointestinal tract, blood vessels, and immune system (53). Given that TWIK2 showed no or little conductance in heterologous expression systems, the physiological functions of TWIK2 is poorly understood (54). Interestingly, a recent study demonstrated that pharmacological inhibition or genetic deletion of the TWIK2 channel blocked the activation of NLRP3 inflammasome induced by ATP and thus reduced the release of caspase-1 and IL-1β (27). In contrast, the TWIK2 channel had no effect on the activation of NLRP3 inflammasome activated by imiquimod or nigericin. The TWIK2 channel was mechanistically suggested to cooperate with the P2X7 receptor activated by extracellular ATP, thus mediated potassium efflux required for NLRP3 inflammasome activation.

Furthermore, TWIK2 deletion prevents inflammatory lung injury in sepsis mice (27). Besides TWIK2, THIK1 channel, another member of K2P channels, was recently discovered to play key roles in microglia (52). THIK1 channel was reported to be the main potassium channel expressed in microglia. Pharmacological inhibition or gene knockout of THIK1 depolarizes microglia, decreasing microglial ramification, reducing surveillance function, and IL-1β secretion. This study indicates that THIK1 is necessary for NLRP3 inflammasome activation and immune surveillance in microglia.

## The Mechanisms of Potassium Efflux During NLRP3 Inflammasome Activation

K^+^ efflux is proposed as an important event upstream of NLRP3 inflammasome activation, and the decrease in intracellular K+ can activate the NLRP3 inflammasome; however, the mechanisms of potassium efflux during NLRP3 inflammasome activation is not understood. Macrophages expressing a constitutively active mutant NLRP3 R258W, which could not be suppressed by high extracellular concentrations of potassium, suggests that potassium efflux may be related to NLRP3 protein conformational change (23). Two individual studies show that potassium efflux is essential for NLRP3 and NEK7 interaction, which is an important part of the assembly of the NLRP3 inflammasome, given that the interaction disappears with high extracellular concentrations of potassium (55, 56). These studies suggest that potassium efflux may be closely related to the conformational change of NLRP3 protein and NLRP3-NEK7 interaction during NLRP3 activation, and the underlying mechanism ought to be further investigated. Moreover, K+ efflux might promote NLRP3 activation by mitochondrial dysfunction and mtROS production (57).

## Summary and Outlook

Given the critical role of NLRP3 inflammasome in autoimmune, metabolic, and neurodegenerative diseases and the essential role of potassium efflux in NLRP3 inflammasome activation, it is of great significance to explore the molecular mechanisms underlying potassium efflux during NLRP3 inflammasome activation under different circumstances.

The important role of the P2X7 receptor and GSDMD in immune responses has gained a lot of attention, both academically and industrially (31, 34). An inhibitor JNJ-55308942 targeting the P2X7 receptor is now in phase I clinical study to treat neuroinflammation (58). The role of pannexin-1 in NLRP3 inflammasome activation following caspase-11 or caspase-8 induced pyroptosis is still under debate. Furthermore, although crystalline substances also depend on potassium efflux to activate NLRP3 inflammasome, this process’s mechanism is not clear and needed to be resolved in the future.

Last but not least, the lately identified TWIK2 and THIK1 channels were the only “specific” potassium channels involved in NLRP3 inflammasome activation (27, 52). Both TWIK2 and THIK1 channels could be attractive therapeutic targets for the treatment of NLRP3 inflammasome related autoimmune diseases in the future.

## Author Contributions

All authors listed have made a substantial, direct, and intellectual contribution to the work, and approved it for publication.

## Funding

This study was supported by research grants from the National Natural Science Foundation of China (81973333), the Young Scholars of Pearl River in Guangdong Province, and the Natural Science Foundation of Guangdong Province (2019A1515010964) to PZ.

## Conflict of Interest

The authors declare that the research was conducted in the absence of any commercial or financial relationships that could be construed as a potential conflict of interest.
